# Oral findings in intensive care unit patients

**DOI:** 10.4317/medoral.27639

**Published:** 2026-04-19

**Authors:** Weslay Rodrigues-da-Silva, Ana Paula Veras Sobral, Hugo Angelo Gomes-de-Oliveira, Aylanne Xavier-de-Lacerda Cavalcante Timoteo Briano, Antonio Carlos Moura Melo Albuquerque, Luciana Ferraz Gominho, Lúcia-de-Fátima Santos Cavalcanti, Ully Dias Nascimento Távora Cavalcanti, Kaline Romeiro

**Affiliations:** 1 Department of Oral and Maxillofacial Pathology, School of Dentistry, Postgraduation Program in Dentistry, University of Pernambuco, Recife, Brazil; 2 Oswaldo Cruz University Hospital (OCUH), Integrated Anatomic Pathology Centre, Recife, Brazil; 3 Department of Clinical and Preventive Dentistry, Federal University of Pernambuco (UFPE), Recife, Brazil; 4 Department of Oral Medicine, Royal Portuguese Beneficent Hospital in Pernambuco, Recife, Brazil; 5 Department of Restorative Dentistry, Federal University Paraíba (UFPB), João Pessoa, Brazil; 6 Pelópidas Silveira Hospital, Division of Dentistry, Recife, PE, Brazil; 7 Postgraduate Program in Dentistry, University of Grande Rio (UNIGRANRIO), Rio de Janeiro, Brazil; 8 Department of Restorative Dentistry, Faculty of Dentistry, Facol University Centre (UNIFACOL), Vitória de Santo Antão, PE, Brazil

## Abstract

**Background:**

The aim of this study was to evaluate oral changes in patients admitted to an intensive care unit (ICU).

**Material and Methods:**

Retrospective, cross-sectional, descriptive study. The following data were collected at a high-complexity referral centre in northeastern Brazil between 2021 and 2024: gender, age, ventilatory support, dental condition, and type of oral change.

**Results:**

A total of 2,692 patients admitted to the ICU were evaluated; 52.1% of these patients were male and the mean age was 69.98 years (±19.53). Oral changes were identified in 58.41% of the patients, with odontogenic infections being the most prevalent (61.49%), followed by clinical conditions suggestive of salivary changes (15.05%) and soft-tissue lesions (9.85%). The presence of oral changes was significantly associated with males (p &lt;0.001) aged from 50 to 59, and from 60 to 69 years (p&lt;0.001), ventilatory support (p=0.037), being partially dentate (p&lt;0.001), and the number of teeth (p&lt;0.001).

**Conclusions:**

These data permit a characterization of the oral health status of this patient profile, reinforcing the importance of the dentist as a member of the multidisciplinary team.

## Introduction

The Intensive Care Unit (ICU) is a specialized area for critically ill patients who require a greater degree of healthcare (1 , 2). ICU patients require multidisciplinary care as their critical condition and the invasive procedures increase stress and hinder management, often leading to oral care being neglected (3 - 5). Most ICU patients exhibit oral lesions and infectious foci prior to admission, including dental caries, residual roots, periodontal disease and gingivitis (6). Furthermore, these patients may develop oral changes resulting from the use of medication secondary to systemic changes and/or ventilatory support, which may involve different tissue types such as the mucosa, bones, teeth and salivary glands (1 , 3 , 7 - 10).

The demographic profile and the frequency of oral changes observed have been well delineated for different groups of patients, including the elderly, patients in nursing homes and those hospitalized in areas other than the ICU (11 - 14). However, information regarding the frequency of oral changes in ICU patients is limited (1 , 15 , 16). Such studies are important since they provide reliable data on the main diagnoses that affect this population (6 , 9), in addition to contributing to the development of prevention and treatment strategies (15). Oral health is often neglected in the ICU where the priority is the maintenance of vital functions. However, oral conditions can have a significant impact on patients' recovery and quality of life. Considering the scarcity of data on this topic, the present study aimed to evaluate the clinical and demographic characteristics of patients presenting with oral changes upon admission to the ICU of a referral centre in Northeastern Brazil.

## Material and Methods

Study design and ethical issues

This retrospective, cross-sectional study investigated the occurrence of oral changes diagnosed in patients admitted, between 2021 and 2024, to the ICU of the Real Hospital Português de Beneficência, located in the state of Pernambuco, Brazil. This study was approved by the Institutional Review Board (Protocol number: 5.727.884) and complies with the Declaration of Helsinki. The study followed the guidelines established by the Declaration of Helsinki for research involving human subjects, as well as the STROBE guidelines for observational studies.

Sample

Patients under the age of 18, those hospitalized for less than 24 hours and individuals who could not be moved on medical grounds, as well as patients with systemic instability or any other condition that might compromise examination, were excluded.

Demographic data collection

Demographic and clinical data were collected from the patients' medical records (gender, age and type of ventilatory support). The type of ventilatory support was categorized as follows: spontaneous breathing, use of a nasal catheter, use of a mask, orotracheal intubation, or tracheostomy.

Clinical examination and Bias

All patients underwent routine oral examination by two experienced dentists using personal protective equipment, with artificial lighting, tongue depressors and sterile gauze. Thorough oral examination included the oral mucosa regions and the teeth. In addition, the teeth were counted and the patients were classified as edentulous, toothed or partially edentulous. The use of a prosthesis was also considered. To minimize bias, two examiners were calibrated with 20 images of varying oral conditions. Patients were assessed independently, with inter-examiner agreement (Kappa&gt;0.68) indicating adequate reliability.

Oral status

Oral changes were identified and classified according to aetiology, using an adaptation of the method proposed by Eduardo et al. (15): Odontogenic infections, any changes of endodontic and/or gingival/periodontal origin; clinical conditions suggestive of salivary changes. This category included lip dryness and oral mucosal dryness; soft-tissue lesions, changes in mucosal integrity of non-infectious origin such as traumatic ulcers, thermal burns, mucositis (associated with oncological treatment), and graft-versus-host disease; non-odontogenic infections, clinical appearance of lesions suggesting a fungal (candidiasis), viral or bacterial infection. Viral infections were classified as recurrent herpes simplex and herpes zoster, while bacterial infections were classified as bullous impetigo; vascular disorders - this category included petechiae and ecchymosis. Bleeding due to trauma was not deemed to be a vascular disorder. The DIOX® portable X-ray machine (MicroImaging, Brazil) was used to better diagnose tooth fracture/dental care and periapical abscesses.

Statistical Analysis

Descriptive and quantitative data analyses were performed using the Statistical Package for the Social Sciences for Windows, v.20.0 (SPSS, Inc., Chicago, IL, USA). Pearson's chi-square test was applied to evaluate the association between categorical variables, the strength of the association being obtained through the odds ratio (OR), and the respective confidence intervals. The Mann-Whitney test was used to compare two categories of numerical variables. A pvalue 0.05 was adopted.

## Results

**Figure 1:**
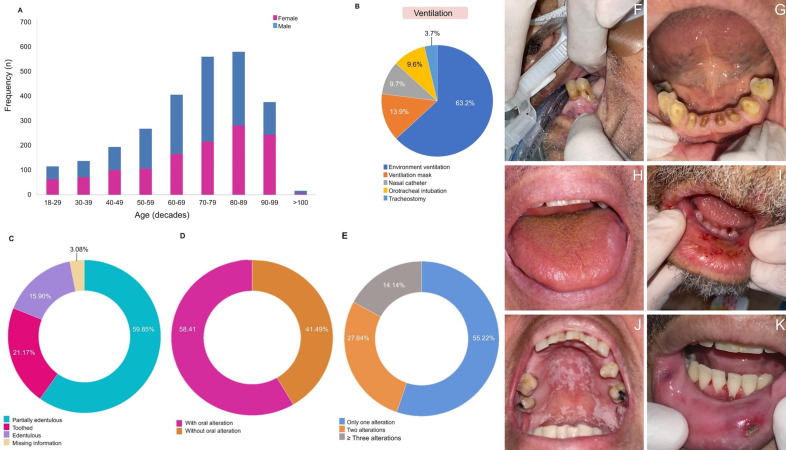
Demographic profile of ICU patients, distribution of oral characteristics, and oral changes. (A) Distribution of cases of ICU patients according to the age and gender. (B) Distribution of cases according to the type of ventilation support. (C) Distribution according to dental characteristics. (D) Distribution of patients according to the presence or absence of oral alteration. (E) Distribution according to the number of oral changes found. (F) Odontogenic infection of gingival/periodontal origin. (G) Odontogenic infection of endodontic origin. (H) Clinical conditions suggestive of salivary changes: oral mucosa dryness. (I) Soft tissue lesion: traumatic ulcer on the lower lip. (J) Non-odontogenic infection caused by Candida, classified as pseudomembranous candidiasis. (K) Vascular disorder characterizing intraoral petechia.

A total of 103 patients were excluded from the study at the request of a physician, due to systemic instability. A total of 2,692 patients were included in the study, having been admitted to the general ICU within the preceding 24 to 48 hours. The age distribution (decades) and gender of individuals admitted to the ICU are shown in Figure 1A. The mean age of the patients was 69.98 years (±19.53). Ages ranged from 18 to 107 years. Most patients were between the 8th and 9th decades of life. A total of 1,402 (52.1%) patients were men, while 1,290 (47.9%) were women (male-to-female ratio of 1.08:1). The distribution of the types of ventilation support is shown in Figure 1B. Most patients did not require ventilatory support, remaining on environmental ventilation (63.2%) (Figure 1B). As far as dental characteristics are concerned, most patients were partially edentulous (59.85%) (Figure 1C).

1

Of the total number of patients evaluated, oral changes were observed in 1,575 (58.41%) (Figure 1D) and a total of 2,597 oral changes were observed (Table 1). Of the total number of patients exhibiting oral changes, 847 (55.22%) exhibited just one oral change (Figure 1E). The most common changes were odontogenic infections (61.49%), followed by clinical conditions suggestive of salivary changes (15.05%) and soft-tissue lesions (9.85%) (Table 1). Among odontogenic infections, 32.49% were of gingival/periodontal origin (Figure 1A) and 28.99% of endodontic origin (Figure 1B). Clinical conditions suggestive of salivary changes were responsible for 15.05% of the oral changes (Figure 1C). Among soft-tissue lesions (9.85%), traumatic ulcers were the most common (9.06%) (Figure 1D). Of the clinically diagnosed, non-odontogenic infections (9.54%), the most common was oral candidiasis (9.25%) (and this was the only type of fungal infection detected) (Figure 1E); however, there were also cases of herpes zoster (0.04%) with oral manifestation, and bullous impetigo (0.04%). Vascular disorders (4.04%) were the least common types of oral change (Figure 1F).

Table 1A statistically significant association was found between the presence of oral changes and the male gender (p&lt;0.001), with ages ranging from 50 to 59 and from 60 to 69 years (p&lt;0.001), on ventilatory support (p=0.037), and partially toothed (p&lt;0.001) (Table 2). It was observed that the use of prostheses was more frequent in patients with oral disorders, although there was no statistically significant association (Table 2). As for the presence of oral changes and the number of teeth, we found a statistically significant association (p&lt;0.001).

Table 2

## Discussion

Information concerning the frequency of oral changes in this patient profile is important since it contributes to the diagnosis and prevention of oral lesions. This article reports on the largest series of patients admitted to an ICU for whom oral changes were evaluated. In terms of gender, our results indicate a male-to-female ratio of 1.08:1. However, a statistically significant association was observed between the presence of oral changes and the male gender (p&lt;0.001). Martins et al. (1) reported a higher prevalence of female patients in the ICU, but oral changes mainly affected male patients. It is believed that males make less frequent use of health services than females. Thus, the low frequency of these changes in females may be related to a history of more frequent healthcare attendance and better oral hygiene practices (8).

Patients admitted to the ICU are generally older than 50 (17). In the present study, the mean age of the patients was 69.98 years (±19.53). Aging may be a risk factor for oral changes since older patients often require multidrug therapy that can reduce saliva production. The repair process also undergoes change with advancing age, which may reduce the intensity and effectiveness of the immune response (13). We found a statistically significant association between oral changes and the 50 to 59 and 60 to 69 age groups (p&lt;0.001).

Odontogenic infections accounted for 61.49% of oral changes detected in the present study. It is known that these infections are mainly caused by microbial biofilm (7 , 18). Although dentists play a key role in diagnosing and treating oral conditions in the ICU, oral hygiene is typically managed by nurses, which is essential for the prevention of infection (1 , 3 , 10). Despite a decline in dental treatments, the rate of tooth extraction continues to be high in Brazil (19). This tooth loss can be observed in our sample in which only 570 (21.2%) patients possessed a full set of teeth, while 1,611 (59.8%) patients had lost at least one tooth prior to being admitted to the ICU. Our results revealed a statistically significant association of oral changes with the number of teeth (p&lt;0.001) and the mean number of teeth (p&lt;0.001). Fractured teeth may cause mucosal trauma. In addition, teeth and their supporting structures retain biofilm, promoting infectious processes. Oral hygiene protocols must therefore be developed for these patients (18).

Ventilatory support, especially prolonged use thereof, is a risk factor for oral soft-tissue lesions due to mucosal dryness and increased microbial growth (5). In the present study, 36.8% of the patients required some type of ventilatory support, which was significantly associated with the presence of oral changes (p=0.037).

The orotracheal tube is one of the medical devices most associated with pressure injuries (8 , 17). The intubation process is considered one of the main causes of trauma in the ICU and a significant contributing factor to the development of these injuries (5 , 15). In our sample, traumatic ulcers were the most common soft-tissue lesion (9.06%). Other tissue lesions such as mucositis (0.73%) were also observed. Cancer patients are at greater risk of developing mucositis as a result of antineoplastic treatment, however, treatment is discontinued as a matter of course when these patients are admitted to the ICU. Silva et al. (10) observed a higher incidence of these lesions (20%) among these patients. These divergent findings might be related to the fact that cancer was one of the least common causes of ICU admission in our study.

Being on ventilatory support facilitates microbial growth and increases the risk of infections such as nosocomial and ventilator-associated pneumonia due to pathogen migration to the respiratory tract (20 , 21). Dry mouth, aspiration, microbiota imbalance and oropharyngeal changes can contribute to pneumonia. Oral candidiasis was found in 9.25% of cases, associated with factors like immunosuppression, malnutrition, aging, endocrine disorders, corticosteroid use and antibiotics (22 - 24). In ICU patients, fungal infections can originate in the oral cavity, worsen the clinical condition, and potentially lead to death. Oral hygiene is essential for preventing such infections (20).

Mucosal dryness is a common finding in patients admitted to the ICU and may be a consequence of reduced salivary flow and/or sialochemical changes secondary to medication use, stress and dehydration (9 , 25 , 26). Dry mouth represented 7.97% of oral changes in the sample. Lip dryness, often caused by environmental factors in the ICU, such as low temperatures from the air conditioning, can be prevented and treated (27). Lip dryness was observed in 7.08% of patients. Besides causing pain, it can compromise the skin barrier and serve as an entry point for secondary infections (5 , 9).

The heterogeneity of the population in respect of age, the reason for ICU admission, ventilatory support and dental condition, represents a limitation of the study. Additionally, difficulties in evaluating the oral cavity of some patients, especially those who were intubated or had involuntary muscle contractions, may have led to underreporting of conditions such as caries and periodontal disease. Longitudinal studies are recommended that include the analysis of confounding factors.

## Conclusions

Oral changes, in particular odontogenic infections, were common in ICU patients. They were more frequent in males aged 50-69 who were on ventilatory support, who were partially dentate, wore dentures or had more teeth. These findings highlight the need for thorough oral examinations by qualified professionals and reinforce the need for the inclusion of dentists in the ICU team.

## Figures and Tables

**Table 1:** Table Frequency and type of oral changes in ICU patients.

Type of oral change	n (%)
Odontogenic infection	1597 (61.49)
Infections of gingival/periodontal origin	844 (32.49)
Gingivitis	790 (30.39)
Tooth mobility	50 (1.92)
Periodontal abscess	3 (0.11)
Pericoronitis	1 (0.03)
Infections of endodontic origin	753 (28.99)
Tooth fracture/dental caries	467 (17.99)
Residual root	279 (10.74)
Periapical abscess	7 (0.26)
	
Clinical conditions suggestive of salivary changes	391 (15.05)
Oral mucosa dryness	207 (7.97)
Dry lips	184 (7.08)
	
Soft-tissue lesions	256 (9.85)
Traumatic ulcer	235 (9.06)
Mucositis	19 (0.73)
GvHD*	1 (0.03)
Thermal burns	1 (0.03)
	
Non-odontogenic infections	248 (9.54)
Fungal Infection (Oral candidiasis)	240 (9.25)
Pseudomembranous candidiasis	144 (5.57)
Denture stomatitis	26 (1.00)
Linear gingival erythema	23 (0.88)
Angular cheilitis	19 (0.73)
Median rhomboid glossitis	18 (0.69)
Acute atrophic candidiasis	10 (0.38)
Viral infection	7 (0.26)
Recurrent labial herpes simplex	6 (0.23)
Oral herpes zoster	1 (0.03)
Bacterial infection	1 (0.03)
Bullous impetigo	1 (0.03)
	
Vascular disorders	105 (4.04)
Petechiae/ecchymosis	105 (4.04)
Total	2597 (100.00)

1

**Table 2:** Table Association of the presence of oral changes with gender, age group, use of prosthesis, ventilatory support and dental condition.

Clinical and demographic characteristics	Oral change		Total	OR (IC 95%)	p-value
	Yes	No			
	n (%)	n (%)	n (%)		
Gender					p(1)<0.001*
Male	862 (61.5)	540 (38.5)	1,402 (100.0)	1.5 (1.3 to 1.7)	
Female	672 (52.1)	618 (47.9)	1,290 (100.0)	1.0	
					
Age groups					p(1)<0.001*
≤39	126 (49.2)	130 (50.8)	256 (100.0)	1.0	
40 to 49	102 (52.6)	92 (47.4)	194 (100.0)	1.1 (0.8 to 1.7)	
50 to 59	171 (62.6)	102 (37.4)	273 (100.0)	1.7 (1.2 to 2.5)	
60 to 69	256 (62.3)	155 (37.7)	411 (100.0)	1.7 (1.2 to 2.3)	
70 to 79	324 (57.9)	236 (42.1)	560 (100.0)	1.4 (1.1 to 1.9)	
80 to 89	353 (59.8)	237 (40.2)	590 (100.0)	1.5 (1.1 to 2.1)	
≥90	202 (49.5)	206 (50.5)	408 (100.0)	1.0 (0.7 to 1.4)	
					
Prosthesis					p(1)=0.126
Yes	616 (55.2)	499 (44.8)	1,115 (100.0)	1.0	
No	918 (58.2)	659 (41.8)	1,577 (100.0)	1.7 (1.5 to 2.1)	
Total	1,534 (57.0)	1,158 (43.0)	2,692 (100.0)		
					
Ventilation					p(1)=0.037*
Ventilation mask	208 (55.8)	165 (44.2)	373 (100.0)	1.0	
Nasal catheter	147 (56.3)	114 (43.7)	261 (100.0)	1.0 (0.7 to 1.4)	
Orotracheal intubation	156 (60.5)	102 (39.5)	258 (100.0)	1.2 (0.9 to 1.7)	
Tracheostomy	71 (71.0)	29 (29.0)	100 (100.0)	1.9 (1.2 to 3.1)	
Total	592 (58.7)	410 (41.3)	992 (100.0)		
					
Dental condition					p(1)<0.001*
Partially toothed	1,092 (67.8)	519 (32.2)	1,611 (100.0)	6.5 (5.1 to 8.3)	
Fully toothed	282 (49.5)	288 (50.5)	570 (100.0)	3.0 (2.3 to 4.0)	
Edentulous	105 (24.5)	323 (75.5)	428 (100.0)	1.0	
Total Group	1,479 (56.7)	1,130 (43.3)	2,609 (100.0)		

2

## Data Availability

Declared none.

## References

[1] Martins HDD, Sales RC, Medeiros DSBD, de Aquino Martins ARL, Lopes MLD de S, Lima KC (2022). Risk factors for oral alterations in intensive care unit patients: A pilot cohort study. J Oral Pathol Med.

[2] Silva AF, Robazzi MLDCC, Dalri RDCDMB, Silveira-Monteiro CA, Mendes AMOC (2019). Presenteeism in multiprofessional team workers in the Adult Intensive Care Unit. Rev Bras Enferm.

[3] Türk G, Kocaçal Güler E, Khorshid L (2012). Oral care practices of intensive care nurses: A descriptive study. Int J Nurs Pract.

[4] Puntillo K, Nelson JE, Weissman D, Curtis R, Weiss S, Frontera J, Gabriel M, Hays R, Lustbader D, Mosenthal A, Mulkerin C, Ray D, Bassett R, Boss R, Brasel K, Campbell M (2014). Palliative care in the ICU: relief of pain, dyspnea, and thirst-A report from the IPAL-ICU Advisory Board. Intensive Care Med.

[5] Jang CS, Shin YS (2016). Effects of combination oral care on oral health, dry mouth and salivary pH of intubated patients: A randomized controlled trial. Int J Nurs Pract.

[6] Steinle EC, Pinesso JAM, Bellançon LB, de Paula Ramos S, Seixas GF (2023). The association of oral health with length of stay and mortality in the intensive care unit. Clin Oral Investig.

[7] da Cruz MK, de Morais TMN, Trevisani DM (2014). Clinical assessment of the oral cavity of patients hospitalized in an intensive care unit of an emergency hospital. Rev Bras Ter Intensiva.

[8] Celik GG, Eser I (2017). Examination of intensive care unit patients’ oral health. Int J Nurs Pract.

[9] Quintanilha RMC, Pereira MRR, de Oliveira SP, Penoni DC, Salgado DR, Agostini M, Torres SR (2024). Oral clinical findings and intensive care unit prognostic scores. BMJ Support Palliat Care.

[10] Silva AP, Caruso P, Jaguar GC, Carvalho PAG, Alves FA (2014). Oral evaluation and procedures performed by dentists in patients admitted to the intensive care unit of a cancer center. Support Care Cancer.

[11] Neto AC, De Paula Ramos S, Sant’ana A, Passanezi E (2011). Oral health status among hospitalized patients. Int J Dent Hyg.

[12] Wong FMF, Ng YTY, Leung WK (2019). Oral Health and Its Associated Factors Among Older Institutionalized Residents-A Systematic Review. Int J Environ Res Public Health.

[13] Gao SS, Chu CH, Young FYF (2020). Oral Health and Care for Elderly People with Alzheimer’s Disease. Int J Environ Res Public Health.

[14] Manninen S, Snäll J, Puolakkainen T, Haapanen A (2025). Severe odontogenic infections in patients with mental disorders: the challenge of ineffective initial treatment. Oral Surg Oral Med Oral Pathol Oral Radiol.

[15] Eduardo FP, Bezinelli LM, Gobbi MF, Bergamin LG, de Carvalho DLC, Corrêa L (2022). Oral lesions and saliva alterations of COVID-19 patients in an intensive care unit: A retrospective study. Spec Care Dentist.

[16] Batista AAF, Ramos KPP, Prado LFA, Araújo AA de S, Martins-Filho PR, Nunes PS (2022). Oral lesions in patients with COVID-19 hospitalized in an intensive care unit: a case-series study. Braz Oral Res.

[17] Galetto SGS, do Nascimento ERP, Hermida PMV, Busanello J, de Malfussi LBH, Lazzari DD (2021). Medical device-related pressure injuries in critical patients: prevalence and associated factors. Rev Esc Enferm USP.

[18] Emery KP, Guido-Sanz F (2019). Oral care practices in non-mechanically ventilated intensive care unit patients: An integrative review. J Clin Nurs.

[19] dos Santos JL, Ferreira RC, Amorim L de P, Santos ARS, Chiari APG, Senna MIB (2021). Oral health indicators and sociodemographic factors in Brazil from 2008 to 2015. Rev Saude Publica.

[20] Araújo ECF, da Silva RO, Raymundo MLB, Vieira TI, de Sousa SA, Santiago BM, Cavalcanti YW (2023). Does the presence of oral health teams influence the incidence of ventilator-associated pneumonia and mortality of patients in intensive care units? Systematic review. Spec Care Dentist.

[21] Zaragoza R, Vidal-Cortés P, Aguilar G, Borges M, Diaz E, Ferrer R, Maseda E, Nieto M, Nuvials FX, Ramirez P, Rodriguez A, Soriano C, Veganzones J, Martín-Loeches I (2020). Update of the treatment of nosocomial pneumonia in the ICU. Crit Care.

[22] Millsop JW, Fazel N (2016). Oral candidiasis. Clin Dermatol.

[23] Uppal HS, De R, D’Souza AR, Pearman K, Proops DW (2003). Bilateral submandibular duct relocation for drooling: an evaluation of results for the Birmingham Children’s Hospital. Eur Arch Otorhinolaryngol.

[24] Xiao Y, Yuan P, Sun Y, Xu Y, Deng X, Wang X, Liu R, Chen Q, Jiang L (2022). Comparison of topical antifungal agents for oral candidiasis treatment: a systematic review and meta-analysis. Oral Surg Oral Med Oral Pathol Oral Radiol.

[25] Berti-Couto SA, Couto-Souza PH, Jacobs R, Nackaerts O, Rubira-Bullen IRF, Westphalen FH, Moysés SJ, Ignácio SA, da Costa MB, Tolazzi AL (2012). Clinical diagnosis of hyposalivation in hospitalized patients. J Appl Oral Sci.

[26] Mohammed A (2014). Update knowledge of dry mouth- A guideline for dentists. Afr Health Sci.

[27] Kim CH, Kim MS, Kang MJ, Kim HH, Park NJ, Jung HK (2019). Oral mucosa pressure ulcers in intensive care unit patients: A preliminary observational study of incidence and risk factors. J Tissue Viability.

